# One- versus 2-day aspirin desensitization in aspirin exacerbated respiratory disease: A quality improvement project

**DOI:** 10.1016/j.jacig.2023.100158

**Published:** 2023-07-26

**Authors:** Emily Gansert, Dan Morgenstern-Kaplan, Angela M. Donaldson, Matthew A. Rank, Alexei Gonzalez-Estrada

**Affiliations:** a Division of Pulmonary, Allergy, and Sleep Medicine, Department of Medicine, Mayo Clinic, Jacksonville, Fla; b Department of Otorhinolaryngology (ENT)/Head and Neck Surgery, Mayo Clinic, Jacksonville, Fla; c Division of Allergy, Asthma, and Clinical Immunology, Department of Medicine, Mayo Clinic, Scottsdale, Ariz

**Keywords:** Aspirin-exacerbated respiratory disease, aspirin desensitization, quality improvement, nonsteroidal anti-inflammatory drugs

## Abstract

**Background:**

Current aspirin desensitization protocols for aspirin-exacerbated respiratory disease (AERD) require from 1 to 3 days to complete.

**Objective:**

Our aim was to assess the implementation of a 1-day versus 2-day aspirin desensitization protocol in patients with aspirin-exacerbated respiratory disease.

**Methods:**

We used a preintervention-postintervention quality improvement design to compare the completion rates, reaction rates, and estimated costs of a 2-day versus 1-day aspirin desensitization. The cost for each desensitization was estimated on the basis of 2017-2020 US Medicare standards. We included the predesensitization variables for FEV_1_ value, urinary leukotriene E_4_ level, absolute eosinophil count (AEC), and total IgE level for each group.

**Results:**

A total of 15 patients underwent a 2-day aspirin desensitization in the 4-year (2017-2020) preintervention period and were compared with 8 patients who underwent a 1-day aspirin desensitization in the 1-year (2021) postintervention period. The desensitization completion rate (93% vs 100% [*P* = 1]) and the mean number of reactions requiring intervention during the desensitization protocols (0.26 vs 0.8 [*P* = .14]) were similar between groups. The average time frame between last polypectomy and desensitization was longer in the 2-day group (1946 vs 39.2 days [*P* = .03]). The mean values for FEV_1_ level, urinary leukotriene E_4_ level, absolute eosinophil count, and total IgE level were 76% vs 83% (*P* = .6), 1084 vs 385 pg/mg (*P* = .2), 686 vs 306 cells/μL (*P* = .74), and 735 vs 278 kU/L (*P* = .5), respectively. The estimated direct cost reduction was $762 per aspirin desensitization for using 1-day vs 2-day aspirin desensitization.

**Conclusion:**

Compared with a 2-day protocol, the implementation of a 1-day aspirin desensitization was characterized by similar completion and reaction rates as well as lower costs.

## Introduction

Aspirin-exacerbated respiratory disease (AERD) is a chronic inflammatory condition that is a progression of asthma to chronic rhinosinusitis with nasal polyps and, ultimately, an intolerance of aspirin or nonsteroidal anti-inflammatory drugs.^1^, ^2^, ^3^ AERD is diagnosed and treated in a stepwise approach that includes medical and surgical intervention. Medical intervention may include aspirin challenge and desensitization.^4^ Following aspirin desensitization, long-term aspirin therapy is effective and safe in as many as 87% of patients.^3^ Current desensitization protocols range from 1 to 3 days to complete. These protocols are often time- and resource-intensive for both the patient and the medical facility.^5^ This study aimed to assess the implementation of a 1-day versus 2-day aspirin desensitization protocol in patients with AERD and determine the most effective, safest, and most efficient protocol.

A preintervention-postintervention quality improvement design was used to compare the completion rates, reaction rates, estimated durations, and estimated costs of a 2-day^6^ versus 1-day^5^ aspirin desensitization protocol for AERD. We included all consecutive adult patients with AERD who were undergoing aspirin desensitization at a single tertiary care center and performed a retrospective chart review with institutional review board approval. The 2-day protocol was derived from Waldram et al.^6^ Day 1 of the protocol utilized nasal ketorolac and oral aspirin, with increasing doses of ketorolac every 30 minutes (1 spray in 1 nostril, 1 spray in both nostrils, 2 sprays in both nostrils, and 3 sprays in both nostrils), 60 mg of aspirin 1 hour after the last spray, and a final 60-mg aspirin dose with a 3-hour observation period. Day 2 of the protocol included a 150 mg of aspirin followed by a 325-mg aspirin dose after 3 hours and a 3-hour observation period. Our 1-day protocol was adapted from DeGregorio et al.^5^ Our protocol included increasing doses of oral aspirin (40.5 mg, 81 mg, 162 mg, and 325 mg) every 90 minutes with a 150-minute observation period.

The preintervention period lasted from November of 2017 through December of 2020, and the postintervention period lasted from January through December of 2021. Success of the aspirin desensitization was defined by tolerance of a 325-mg aspirin dose. All patients then began taking aspirin, 650 mg twice a day, with plans to decrease the dose during a 3-month follow-up period. The cost of each desensitization was estimated on the basis of the averaged 2017-2020 US Medicare standards. The predesensitization variables for prebronchodilator (FEV_1_) value, urinary leukotriene E_4_ (LTE_4_) level, absolute eosinophil count (AEC), total IgE level, number of endoscopic sinus operations before desensitization, and time from the last endoscopic sinus operation to desensitization were included for each group. Because of the coronavirus disease 2019 (COVID-19) pandemic, peak flow use, spirometry, and pulmonary function tests were suspended; as a result, not every patient underwent an airway limitation test before aspirin desensitization. We based asthma control on clinical presentation. All patients undergoing desensitization had their asthma optimized. Descriptive statistics were performed for all variables. Categoric variables were summarized as frequencies and percentages, and numeric variables were summarized as means and SDs. To determine statistical difference between groups, we used the Fisher exact test for categoric variables and Mann-Whitney *U* tests for numeric variables. The statistical test was 2-sided, and statistical analysis was performed in Microsoft Excel and R software. Statistical significance was determined by a *P* value less than .05.

## Results and discussion

A total of 23 patients with AERD were included in this study; their mean age was 54.4 years (SD = 14.74 years), 52% were female, and 87% were non-Hispanic. In all, 15 patients underwent a 2-day aspirin desensitization during the preintervention period and were compared with 8 patients who underwent a 1-day aspirin desensitization in the postintervention period (Table I). In both groups, the majority of the patients were never-smokers (73% vs 63%), had asthma (100%), had chronic rhinosinusitis with nasal polyps (87% vs 100%), and were overweight. A lower percentage of members of the 2 groups had an adverse reaction to aspirin or a nonsteroidal anti-inflammatory drug (50% vs 47%). There were no statistically significant differences between the predesensitization parameters, which included FEV_1_ value, LTE_4_ level, AEC, and total IgE level. The mean values for FEV_1_ value, LTE_4_ level, AEC, and total IgE level were 76% vs 83% (*P* = .6), 1084 vs 390 pg/mg (*P* = .2), 686 vs 572 cells/μL (*P* = .74), and 735 vs 186 kU/L (*P* = .5). The average time frame between last endoscopic sinus operation and desensitization was longer in the 2-day group (1946 vs 39.2 days [*P* = .03]). The desensitization completion rate (93% vs 100% [*P* = 1]) and the mean number of reactions requiring intervention during the desensitization protocols (.26 vs .8 [*P* = .14]) were similar between groups. One patient who underwent the 2-day protocol did not complete the desensitization on account of an anaphylactic event, and 2 patients who underwent the 2-day protocol required an additional aspirin desensitization because the desensitization had to be held for more than 48 hours, owing to procedures. The average follow-up time in months was longer in the 2-day group (12.7 vs 3.5 months [*P* = .01]). Overall, use of a 1-day protocol resulted in an estimated direct cost reduction of $762 (*P* < .001), and a time reduction of 6 hours per aspirin desensitization (*P* < .001) when compared with the 2-day aspirin desensitization protocol (Table I).

**Table I:** Characteristics of 1-day versus 2-day aspirin desensitization protocols in aspirin exacerbated respiratory disease (n = 23)∗

Characteristic	ASA desensitization protocol		*P* value
	1-day (n = 8)	2-day (n = 15)	
Demographics			
Age (y), mean (SD)	47±16	57±12	.23
Sex, no. (%)			.61
Female	2 (25)	9 (60)	
Male	6 (75)	6 (40)	
Race, no. (%)			1
White	7 (88)	13 (87)	
Non-White	1 (12)	2 (13)	
Baseline characteristics, mean (SD)			
No. of endoscopic sinus operations before diagnosis	3 (2.12)	2.2 (1.61)	.46
No. of endoscopic sinus operations before desensitization	3.8 (1.78)	2.8 (1.56)	.26
Days between last endoscopic sinus operation and desensitization	39.2 (25.9)	1946 (3513)	.03
Percent predicted FEV_1_ (%)	83 (18.3)	76 (16.9)	.6
Urinary LTE_4_ level (pg/mg)	385 (117)	1084 (1354)	.2
Peripheral eosinophil count (cells/μL)	306 (397)	686 (645)	.74
Total IgE level (kU/L)	278 (276)	735 (1028)	.5
ASA desensitization safety and tolerability			
Completed protocol, no. (%)	8 (100)	14 (93.3)	1
Continued ASA after desensitization, no. (%)	5 (63)	12 (80)	1
Need for additional desensitization, no. (%)	0 (0)	2 (13)	1
Follow-up time (mo), mean (SD)	3.5 (2.1)	12.7 (6.5)	.01
ASA desensitization time and cost			
Estimated cost	$833.64	$1597.81	<.001
Estimated time (h)	7	13	<.001

*ASA*, Aspirin; *AERD*, aspirin exacerbated respiratory disease; *CRSwNP*, chronic rhinosinusitis with nasal polyps.

∗ Only 63% of patients with AERD have followed up for the 1-day protocol.

Compared with implementation of a 2-day protocol, implementation of a 1-day aspirin desensitization revealed similar completion and reaction rates, with lower cost, intervention time, and follow-up response time. However, the mean follow-up time and days between last endoscopic sinus operation and desensitization for the 2-day protocol could have been influenced by the COVID-19 pandemic or other factors that could have skewed the results for these variables. Longer time between polypectomy and desensitization can lead to polyp return and an increased polyp burden, which increases the chance of adverse reactions during desensitization. These results were similar to or higher than those of other studies that utilized a 1-day protocol. The 1-day 90-minute protocol used by DeGregorio et al had a 93% completion rate, with 54% of patients requiring 1 or more nebulizer treatments.^5^ The 1-hour dose escalation protocol by Chen et al demonstrated a 40% 1-day and 60% 2-day completion rate, with 86% of patients experiencing a reaction.^7^ A 6.5-hour, 3-dose clinic protocol followed by an at-home escalation to 650 mg twice daily that was conducted by Salgado et al had a 93% in-clinic completion rate and an 81% at-home completion rate.^8^ The limitations of our study include the small sample size, lack of diversity, lack of age adjustment, nonrandomized study population, and low or no documented prior reactions to aspirin or a nonsteroidal anti-inflammatory drug. A population with a higher rate of prior reactions could have allowed further safety evaluation of the 1-day protocol. Monitoring during a 1-day protocol should be the same as that during a 2-day protocol, including asthma optimization before desensitization. Additionally, the costs of desensitization were estimated by averaged Medicare standards rather than by billing records, which would provide more accuracy regarding cost. Overall, a 1-day compared to a 2-day aspirin desensitization reduces the time and health care costs without reducing the quality of care.

## Disclosure statement

Disclosure of potential conflict of interest: The authors declare that they have no relevant conflicts of interest.

**Clinical implications**

In this quality improvement single-center cohort, the tolerability and safety of 1-day versus 2-day aspirin desensitization in patients with AERD were similar.
